# A Secure and Traceable Vehicles and Parts System Based on Blockchain and Smart Contract

**DOI:** 10.3390/s22186754

**Published:** 2022-09-07

**Authors:** Chin-Ling Chen, Zhi-Peng Zhu, Ming Zhou, Woei-Jiunn Tsaur, Chih-Ming Wu, Hongyu Sun

**Affiliations:** 1 School of Information Engineering, Changchun Sci-Tech University, Changchun 130600, China; 2 Department of Computer Science and Information Engineering, Chaoyang University of Technology, Taichung City 413310, Taiwan; 3 School of Computer and Information Engineering, Xiamen University of Technology, Xiamen 361024, China; 4 Computer Center, National Taipei University, New Taipei City 237303, Taiwan; 5 Department of Computer Science and Information Engineering, National Taipei University, New Taipei City 237303, Taiwan; 6 School of Civil Engineering and Architecture, Xiamen University of Technology, Xiamen 361024, China; 7 Department of Computer Science, Jilin Normal University, Siping 136000, China; 8 State Key Laboratory of Numerical Simulation, Siping 136000, China

**Keywords:** blockchain, smart contract, automation supply chain, traceability, asymmetry cryptography

## Abstract

As society advances, so does the total number of vehicles on the road, creating a massive consumer market for automobiles. According to statistics, a major portion of today’s traffic difficulties are caused by accidents caused by subpar cars and auto parts. As a result, each country has, over time, enacted equivalent rules and regulations to prevent such tragedies. However, in the face of profit, some people are desperate enough to employ illegal parts and illegally modified cars, and auto fraud is rampant. As a result, we employ the blockchain of the symmetrical Blockchain’s digital ledger and smart contract technology to build a decentralized supply chain system that can identify specific parts. In this study, we design and discuss the proposed system framework by user functions and the flow of parts based on blockchain, and we discuss communication protocols that use the symmetry and asymmetry cryptography, algorithms, properties, and security of the mechanism while providing related analysis and comparing the properties and costs of the system with other studies. Overall, the proposed method has the potential to successfully address the issue of automobile fraud.

## 1. Introduction

### 1.1. Background

As of 2020, according to the Bureau of Transportation Statistics (BTS) and National Bureau of Statistics of China statistics, the total number of vehicles in the US is about 276 million, and 280 million in China. In 2020, the world car production grew to 76 million [1,2,3].

With that many vehicles, a huge vehicle consumer market is produced, and the same with many traffic problems that are due to the vehicles and parts themselves. For example, in the National Motor Vehicle Crash Causation Survey (NMVCCS) [4], an estimated 44,000 crashes are caused by vehicles, which is about 2% of the crashes counted by NMVCSS; additionally, the National Highway Traffic Safety Administration in the literature [5] stated that the critical causes in 10.5% of crashes are steering, suspension, transmission, and engine failures, while about 21% of crashes are caused by various other vehicle failures or defects. Hoque and Hasan [6] stated that: as a percentage of the total number of crashes, vehicle defects caused 16.0% of the crashes and 29.0% of the total casualties by the same factor. It can be seen that unqualified parts would reduce the stability of the car, and then lead to the occurrence of traffic accidents.

Thus, to reduce traffic problems that are caused by a flaw in vehicles or parts, many countries limit illegally modified vehicles and the sale of non-compliant parts or other car equipment and devices by laws or regulations. For example, in the United States, where car control is relatively loose, California law considers it illegal to sell non-compliant car equipment and devices, and other states have similar laws and regulations [7]. Additionally, under the section 75 of the Road Traffic Act 1988 in the UK, it is an offense to alter a vehicle in such a way that the use of the vehicle on a road would be unlawful [8]. This is the same for other countries in the world, such as Japan or China [9,10].

However, some services such as repair and car maintenance require more professional car knowledge. Although the law regulates the sale of car modifications and parts, car repair frauds are still common, because the notion of every car mechanic or car repair company being honest is unrealistic. For example, in some auto repair shops, the owners use counterfeit auto parts instead of high-quality parts to decrease costs [11], and some auto manufacturers privately allow their automakers to modify vehicles privately [12]. In addition, some dealers also sell accident cars or used cars as new cars after modification to make profits [13]. These defective vehicles will increase the probability of traffic accidents, reducing the trust between consumers and car sales-as-a-service providers. This is very detrimental to the safety of life and property of the market and consumers. 

Therefore, only having legal constraints is not enough. We need to take practical measures to supervise vehicles and parts to ensure the legality and qualification of vehicles and parts on the road. This in turn minimizes consumer exposure to car fraud and curbs illegal car modifications.

Existing supply chain usage generally involves tagging parts using radio-frequency identification (RFID) and one-dimensional or two-dimensional barcodes and then going to a centralized database for information access. Unfortunately, the data in the system can be easily tampered with or falsified, and it is not easy or even possible to trace the flow of parts. A decentralized blockchain-based system, however, is a superior solution to make the information more reliable and is traceable, immutable, secure and transparent. In addition, the Elliptic Curve Digital Signature Algorithm (ECDSA) [14] is used in our system to ensure data integrity and this system is built in Hyperledger Fabric [15].

All in all, in this study, we proposed a based-blockchain system that will accomplish the following:

(1) Ensure data integrity.
(2) Construct a simple quality identification scheme.
(3) Enable traceable, identifiable parts service with efficiency and mutual trust.

### 1.2. Related Works

The automotive supply chain (ASC) has been an intricate system due to the various parts used in each vehicle, the need for many part supplies, and the many stakeholders that exist in the ASC. Before this study, lots of scholars on the issue have also combined blockchain with supply chain, as sown in Table 1.

Chen et al. [16] proposed a relatively complete theoretical framework for blockchain-based supply chains by elaborating on their proposed Supply Chain Quality Management (SCQI) and briefly discussing the issues that arise in the context of the case, but there is no mention of arbitration in the study. Sharma et al. [17] proposed a blockchain-based distributed architecture for the smart city automobile industry that examines the entire process from many perspectives and suggests a practical strategy. However, the research does not elaborate on the circulation process of parts and does not address the algorithms necessary to carry out the suggested circulation process. Kim et al. [18] handle the authentication of genuine vehicle parts via both Blockchain Governance Game (BGG) [19] and Fog Computing [20] techniques. However, the studies lack a thorough examination of the roles of the various blockchain tasks and do not suggest a comprehensive service structure. In the study by Miehle et al. [21], the authenticity and tracking and tracing of the source of parts are addressed, access control and licensing systems to secure private license chains are introduced, archiving using external chains and external databases is enabled, and the entry barrier for SMEs to the alliance chain is lowered, thereby effectively improving the supply chain’s comprehensiveness and integrity, but the regulation and the stalemate are not addressed. Hao developed a Blockchain-based logistics monitoring system (BLMS) in the study [22], which allows customers, logistics operators, and all other parties in the supply chain to track their parcels and information to ensure fairness and transparency, but not enough for the subsequent regulation of automotive services. Yahiaoui’s paper [23] describes a blockchain-based supply chain system and briefly explores the integration of its blockchain supply chain. Li and Ye [24] integrated blockchain technology into the ASC, customizing smart contracts to meet functional requirements, and demonstrating product traceability to consumers and regulators. Wang et al. [25] applied blockchain to auto service to emphasize the importance of component supply chain management, and subsequent service assurance, and offered a blockchain-based Product-Service System (PSS) framework for vehicles and several other application frameworks, but no privacy protection is provided for transactions between supply chain parties, and no specific algorithm or implementation is proposed.

**Table 1:** Comparison of existing auto parts traceability system.

Authors	Year	Objective	Technologies	Merits	Demerits
Chen et al. [16]	2015	A theoretical framework for combining blockchain and supply chain	Blockchain	Proposed intelligent quality management of supply chain based on the blockchain technology.	There is no discussion on the regulation and analysis of services outside the supply chain.
Sharma et al. [17]	2018	a distributed framework model for the entire life cycle phases of the automotive industry blockchain-based	Blockchain	Analyzing the processes of the automotive industry from multiple perspectives and provided a miner node algorithm.	There is no elaboration on the flow process of the parts and no proposed algorithm to be implemented for the flow process.
Kim et al. [18]	2019	A blockchain-based design for authentication of automotive parts	BGG, Fog Computing	Provide service of authentic certification of auto parts and protection of blockchain.	Lack of analysis of the role of stakeholders in the supply chain.
Miehle et al. [21]	2019	A traceable parts supply chain application built on blockchain and smart contracts	Distributed Ledger, Smart Contract, Blockchain	Introduces access control and licensing systems to secure private license chains, and use external chains and external databases to archive.	There is no solution to the regulation of all parties in the supply chain, and there is no corresponding analysis of the subsequent service of the car.
Helo and Hao [22]	2019	A Blockchain-based logistics monitoring system prototype	JavaScript, Blockchain	All parties on the chain can track and access their package information.	No corresponding solution is proposed for the regulation of subsequent car services.
Yahiaoui et al. [23]	2020	Blockchain and smart contract-based supply chain model	Blockchain	An ASC system based on blockchain and smart contracts is proposed and analyzed.	There is no description of the parties of the ASC, algorithms, and car maintenance services.
Li and Ye [24]	2020	Combines blockchain and ASC for distributed storage of production and sales data	Blockchain, Smart Contract	Ensures the security of ASC data, increases the mutual trust of the parties, and increases that process sensitive data.	No analysis is made for the subsequent service of the car, and no specific algorithm is proposed.
Wang et al. [25]	2020	Blockchain-based Product-Service System service framework for vehicle products	Blockchain, smart-contract	All parties to accurately update and verify vehicle information and easier to verify the condition of vehicles in usage.	no specific algorithm or implementation is proposed.

In this paper, we use a symmetrical copy of the decentralized ledger for all users under the security of asymmetric cryptography. the contents of the other sections are as follows: Section 2 involves some related knowledge of this study. Section 3 describes the communication protocol and algorithm of each phase. We analyzed the characteristics and security issues in Section 4. In Section 5, we make some evaluations for communication costs and computation costs. Lastly, we conclude this paper in Section 6.

## 2. Preliminary

### 2.1. Blockchain and Smart Contracts

Blockchain Technology systems came from a paper on the cryptocurrency Bitcoin, “Bitcoin: A Peer-to-Peer Electronic Cash System” [26], proposed by a named Satoshi Nakamoto in 2008. It involves many disciplines, such as mathematics, cryptography, and computer science. In the blockchain, distributed computational storage, public and private keys, real-time broadcasting, and timestamping bring the characteristics of being decentralized, transparently developed, and tamper-proof, and the data structure Merkle tree is used to ensure the traceability of the blockchain. These features make blockchain that can be integrated with various fields.

Smart contacts were proposed by Nick Szabo, a well-known American computer scientist [27]. Smart contacts are codes that run on the blockchain are and automatically executed on the blockchain when conditions are met and cannot be accessed by anyone for execution [28,29]. It is the digital equivalent of traditional contracts, and combined with these blockchains, such as decentralization, tamper-evident, transparent traceability, perpetual operation, and mutual corroboration, smart contracts achieve the effect of decentralization from trusting third-party institutions to trusting the contract itself.

### 2.2. ECDSA

ECDSA was proposed by Rivest et al. It combines Digital Signature Algorithm (DSA) and Elliptic Curve Cryptography (ECC). Compared with traditional encryption methods, ECDSA has the characteristics of smaller parameters, keys and certificates, stronger key bit strength, and faster operating speed [15,30,31,32].

Suppose that A wants to send a message M to B. The signature is generated by sender A and verified by receiver B. Firstly, both parties must agree on the elliptic curve (CURVE, G, *n*), where G is the base point on the curve, *n* is the order of G, and H is the hash function.

Signature: A chooses a random integer $d_{A}$ as a private key with values in the range [0, *n* − 1], and generates the public key $Q_{A}=d_{A}G$.Computing: $z=h\left(m\right)$, $kG=(x_{1},y_{1})$, $r=x_{1}\mathrm{mod}n$ and $s=k^{-1}(z+rd_{A})\mathrm{mod}\;n$. Then, the message *m* and the signature value (r,s) are sent to B.

Verification: B verifies the correctness of the message after receiving the signature value and message m from A. B calculates: $z{}^{\prime }\;=\;h\left(m\right)$, $a_{1}\;=\;z{}^{\prime }s^{-1}\;\mathrm{mod}\;n$, $a_{2}=rs^{-1}\;\mathrm{mod}\;n$, $(x{}^{\prime },y{}^{\prime })\;=\;a_{1}G\;+\;a_{2}Q_{A}$. If the equation $r=x{}^{\prime }\mathrm{mod}n$ holds, the verification passes.

### 2.3. Hyperleader Fabric

Hyperleader Fabric was led by IBM and Linux, a blockchain-based open-source project. It is mainly to establish an enterprise-class distributed ledger system compatible with pluggable consensus mechanisms and supporting identity authentication, which is typical of current federated chains. Additionally, Hyperleader Fabric is modular, scalable, and provides privacy and confidentiality features to enable the platform to give social good, insurance, and finance, as well as supply chain logistics and other industry use cases to provide more effective and novel features.

## 3. Proposed Scheme

**Figure 1:**
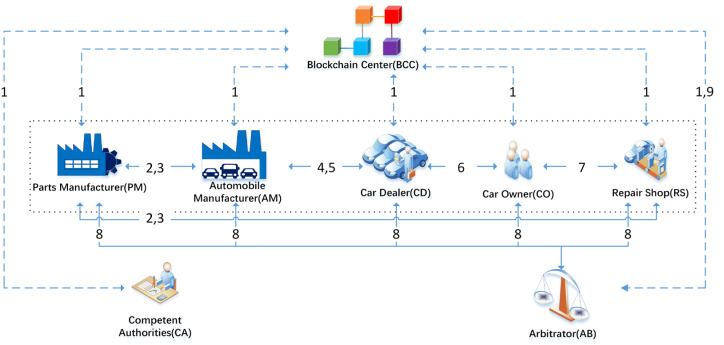
System architecture diagram.

This study uses a symmetrical copy of the blockchain-based ledger technology to build a new automotive parts traceability system by building a Hyperleader Fabric federated chain to implement some functions following text. The system consists of the shareholder’s members of the federated chain Parts Manufacturer (PM), Automobile Manufacturer (AM), Car Dealer (CD), Car Owner (CO), and Repair Shop (RS), as well as Competent Authorities (CA) and Arbitrator (AB) and Blockchain Center (BCC). The system framework is shown in Figure 1.

### 3.1. System Architecture

(1) Parts Manufacturer (PM): PM obtains orders from automobile manufacturers (AM) and Repairers Shop (RS), and then produces the corresponding parts according to the order information and sells them to AM and RS.

(2) Automobile Manufacturer (AM): AM is responsible for the production of research and development of cars, ordering parts from PM for car production. In the meantime, AM also is the seller of car dealers.

(3) Car Dealer (CD): CD is the wholesale vehicle from AM and will sell the vehicle to the consumer (also known as the car owner (CO)).

(4) Car Owner (CO): The end-user of the car, who needs to buy the car from CD, is also the consumer of the Repair Shop (RS) and can go to RS for vehicle repair and parts replacement.

(5) Repair Shop (RS): Order parts from PM to repair the consumer’s vehicle.

(6) Competent Authorities (CA): If a member of the alliance chain is unsure of the legitimate source of a part, the auditor has the right to certify any problems with the flow of the part.

(7) Arbitrator (AB): A third-party arbitrator that receives complaints from members of the alliance chain, can find the flow of parts for cars via the Internet, and can find broken parts that are in circulation on the market.

(8) Blockchain Center (BCC): A blockchain that records key information about parts and vehicles as well as information about the distribution process, and the blockchain associates the ID of the recorded part or vehicle with the vehicle or part. The chain code in the BCC can check the status of the part during the transaction. At the same time, each member needs to register with the blockchain center and request a unique ID to be added to the blockchain.

Figure 1 shows the process of a car part passing through the manufacturer of the part to the car manufacturer, then the car manufacturer agrees to assemble it, then it passes through the dealership, the owner, and through the manufacturer of the part to the repair shop and then to the owner. Of course, in reality, there is more than one member in the alliance chain, and the diagram only shows the flow of parts or cars. And the numbers 1–9 of the Figure 1 is correspond to step 1–9. A description of the specific distribution process is as follows.

Step 1. Each role must register an account on BCC; simultaneously, BBC records the specific information of each member and returns a pair of public and private keys.
Step 2. When AM needs to produce a batch of cars or RS needs to receive a batch of parts, it needs to order parts from PM and send the order information to PM.
Step 3. When PM receives the order information, it will produce the parts and engrave the ID number of each part on the part, and send the parts to AM or RS.
Step 4. If the CD is obtaining a batch of cars from the AM, it needs to send the order information to the AM.
Step 5. AM receives the order and delivers the products to CD.
Step 6. CO goes to CD to buy the vehicle and CO needs to provide the identity for the transaction.
Step 7. CO goes to RS to repair the vehicle.
Step 8. If either party disputes the quality or origin of the parts, they may submit a request for arbitration to the AB.
Step 9. Parts and vehicle-related information and circulation process information are recorded on BCC, AB can retrieve and verify the parts and vehicle-related records through BCC.

### 3.2. Data Definition

**Figure 2:**
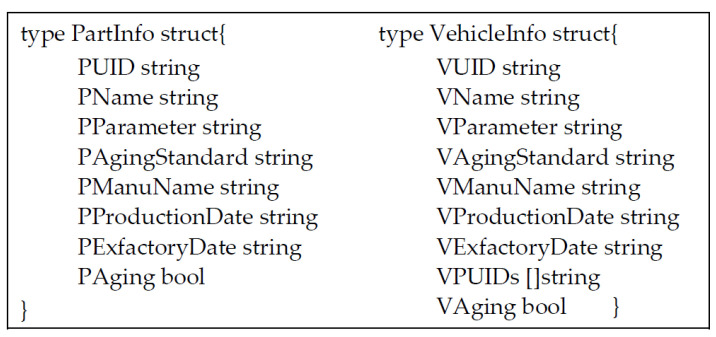
The chaincode structure of the parts and car.

**Figure 3:**
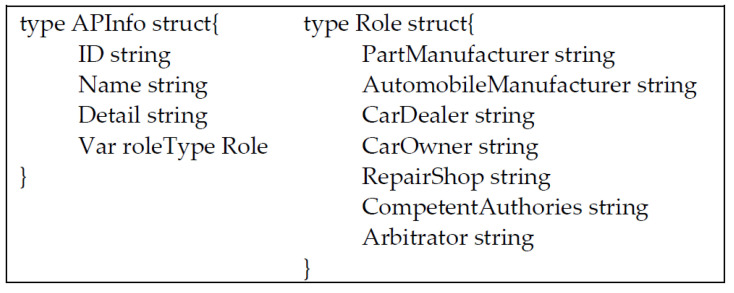
Chaincode structure of the accessing party and the enumeration of the role type.

Figure 2 and Figure 3 are the basic structure of chain code in our designation. Figure 2 shows the product message structure of parts and vehicles. When the product of a vehicle or a part circulates in every Access Party (AP), its details will disclose this structure. In Figure 3, the left shows the storage structure of AP, and the right shows the definition of roles.

### 3.3. Registration Phase

**Figure 4:**
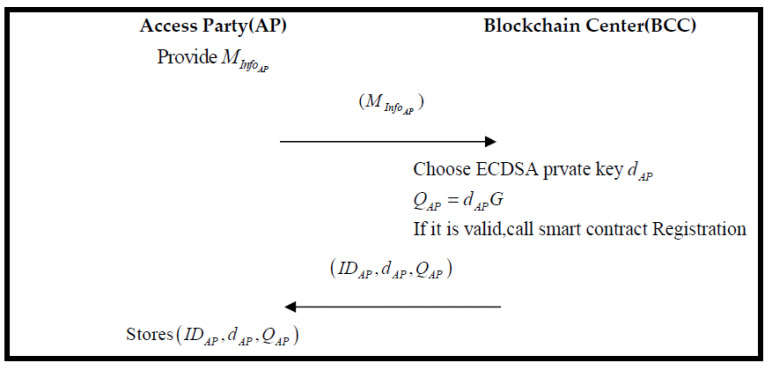
The flowchart of the registration phase.

All parties who join the system must register an account with BCC. When registration is successful, BCC records its message and returns a pair of public key and private key to the member of the register. The specific registration process is shown in Figure 4.

Step 1. AP sends its message $M_{Info_{AP}}$ (e.g., name, role type, etc.) to the blockchain center for the registration request.
Step 2. BCC uses ECDSA to create a private key $d_{AP}$ using the key to calculate the public key $Q_{AP}$:
  (1) $$Q_{AP}=d_{AP}G$$

If the creation is successful, add the role and trigger smart contact. The algorithm of the smart contract is as follows: Algorithm 1. Then, BCC sends $\left(ID_{AP},d_{AP},Q_{AP}\right)$ to AP.

Step 3. AP receive and storage $\left(ID_{AP},d_{AP},Q_{AP}\right)$.

**Algorithm 1:** Chaincode Registration of the proposed scheme.
func Registration (var Name string, var Detail string, var Role string)(UID string){      UID = GenerateUID()      count++      AP[count].UID = UID      AP[count].Name = Name      AP[count].Detail = Detail      AP[count].Role = Role      return UID }

### 3.4. Authentication Phase

**Figure 5:**
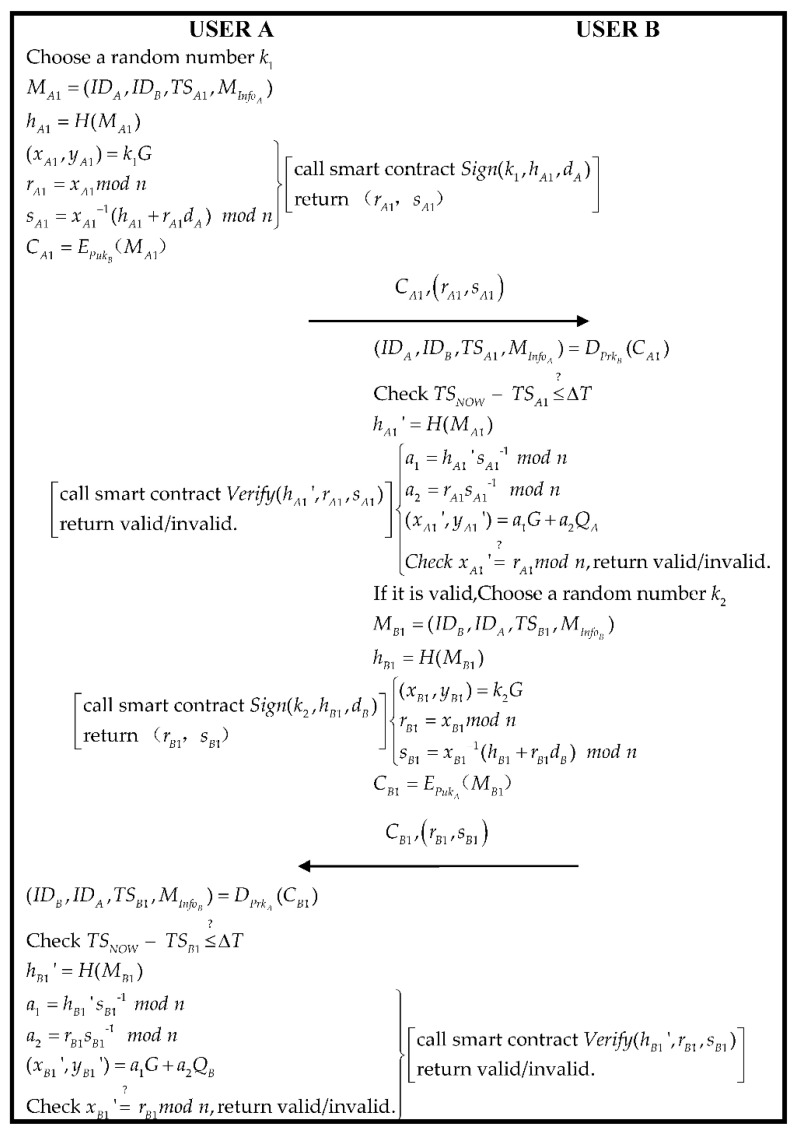
The flowchart of the authentication phase.

Since the actors in the initial stage of the blockchain cannot verify each other’s true identity, both parties who need to perform actions need to be authenticated. The “signature” and “verification” are required when using the algorithm ECDSA implemented for authentication. We assume both users A and B need to authenticate. The specific implementation flow is shown in Figure 5. User A generates a random number $k_{1}$ and a message $M_{A}{}_{1}$ and calculates $h_{A1}$:

(2) $$M_{A}{}_{1}=(ID_{A},ID_{B},TS_{A1},M_{Info_{A}})$$

(3) $$h_{A1}=H(M_{A}{}_{1})$$

Then, User *A* calculates the parameter of ECDSA and through “Sign” of Algorithm 2 generates a signature. The specific process of signature shows in Equations (4)–(6):

(4) $$(x_{A1},y_{A1})=k_{1}G$$

(5) $$r_{A1}=x_{A1}\mathrm{mod}\;n$$

(6) $$s_{A1}=x_{A1}{}^{-1}(h_{A1}+r_{A1}d_{A})\mathrm{mod}\;n$$

Then, *A* uses *B*’s public key $Puk_{B}$ to encrypt a message $M_{A1}$:

(7) $$C_{A1}=E_{Puk_{B}}(M_{A}{}_{1})$$

Finally, A sends the information that is A generating $C_{A1},\left(r_{A1},s_{A1}\right)$ to B.

Step 1. User B receives a message from A and uses B’s private key $Prk_{B}$ deciphering $C_{A1}$ to acquire the data $\left(ID_{A},ID_{B},TS_{A1},Info_{A}\right)$ within the message $M_{A1}$. In the meantime, determine whether the timestamp is legal or not:
  (8) $$\left(ID_{A},ID_{B},TS_{A1},M_{Info_{A}})=D_{Prk_{B}}(C_{A1}\right)$$
  (9) $$TS_{NOW}-TS_{A1}\overset{?}{\leq }\Delta T$$

If Equation (9) is true, the smart contract “*Verify*” of Algorithm 2 will trigger and verify the signature of ECDSA. The specific process of verification is shown in Equations (10)–(14):

(10) $$h_{A1}{}^{\prime }=H(M_{A1})$$

(11) $$a_{1}=h_{A1}{}^{\prime }s_{A1}{}^{-1}\;\mathrm{mod}\;n$$

(12) $$a_{2}=r_{A1}s_{A1}{}^{-1}\mathrm{mod}\;n$$

(13) $$(x_{A1}{}^{\prime },y_{A1}{}^{\prime })=a_{1}G+a_{2}Q_{A}$$

(14) $$x_{A1}{}^{\prime }\overset{?}{=}\;r_{A1}\mathrm{mod}\;n$$

If Equation (14) is true, the message is from A, which can be confirmed. Then, B generates a random number $k_{2}$ and a message $M_{B}{}_{1}$ and calculates $h_{B1}$:

(15) $$M_{B}{}_{1}=(ID_{B},ID_{A},TS_{B1},M_{Info_{B}})$$

(16) $$h_{B1}=H(M_{B1})$$

Then, B calculates the parameter of ECDSA and generates a signature through the “Sign” of Algorithm 2. The specific process of signature is shown in (17)–(19).

(17) $$(x_{B1},y_{B1})=k_{2}G$$

(18) $$r_{B1}=x_{B1}\mathrm{mod}\;n$$

(19) $$s_{B1}=x_{B1}{}^{-1}(h_{B1}+r_{B1}d_{B})\mathrm{mod}\;n$$

Then, B using the public key $Puk_{A}$ of A encrypts a message $M_{B1}$:

(20) $$C_{B1}=E_{Puk_{A}}(M_{B}{}_{1})$$

Finally, B sends information $C_{B1},\left(r_{B1},s_{B1}\right)$ to A.

Step 2. When A receives a message from B, it uses its own private key $Prk_{A}$ to decode $C_{B1}$ and acquire information $\left(ID_{B},ID_{A},TS_{B1},Info_{B}\right)$ within $M_{B1}$. In the meantime, it is verified whether the following timestamp is true or not true:
  (21) $$\left(ID_{B},ID_{A},TS_{B1},M_{Info_{B}})=D_{Prk_{A}}(C_{B1}\right)$$
  (22) $$TS_{NOW}-TS_{B1}\overset{?}{\leq }\Delta T$$

If Equation (22) passes, the smart contract “*Verify*” of Algorithm 2 will trigger and verify the signature of ECDSA. The specific process of verification shows in Equations (23)–(27):

(23) $$h_{B1}{}^{\prime }=H(M_{B1})$$

(24) $$a_{1}=h_{B1}{}^{\prime }s_{B1}{}^{-1}\;\mathrm{mod}\;n$$

(25) $$a_{2}=r_{B1}s_{B1}{}^{-1}\mathrm{mod}\;n$$

(26) $$(x_{B1}{}^{\prime },y_{B1}{}^{\prime })=a_{1}G+a_{2}Q_{B}$$

(27) $$x_{B1}{}^{\prime }\overset{?}{=}\;r_{B1}\mathrm{mod}\;n$$

If Equation (35) passes, we can confirm the message is A sending to B. The authentication between user A and user B is successful.

**Algorithm 2:** Chaincode Sign and Verify the proposed schemefunc Sign(var *k* string, var *h* string, var *d* string){        *(x, y) = k ∗ G*        *r = x % n*        *s = (1/k) ∗ (h + r ∗ d) % n*        return (*r, s*) } func Verify(var h string, var r string){        *a1 = (z/s) % n*        *a1 = (r/s) % n*        *(x, y) = a1 ∗ G + a1 ∗ G*        if *x == r*                return “valid”        else                return “invalid” }

### 3.5. Order and Transaction Phase

**Figure 6:**
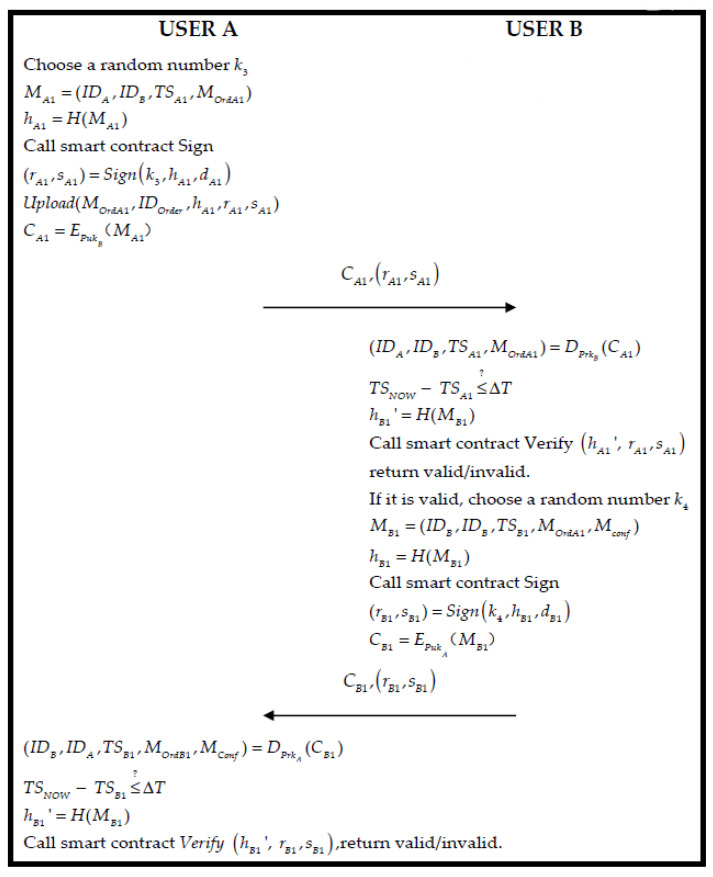
The flowchart of the order phase.

In the phase, we assume both roles that are User A and User B to simulate order and transaction actions. In this phase, A is the buyer purchasing products, and B is the seller. If the AM needs to perform car production and RS is short of parts for vehicle repair and needs to order parts from PM, then User A is AM and RS and User B is PM. If CD needs to order vehicles for sales activities, then User A is CD, and User B is AM at this time. The flowchart is as follows in Figure 6.

Step 1. User A generates a random number $k_{3}$ and message $M_{A}{}_{1}$ and calculates $h_{A}{}_{1}$:

(28) $$M_{A}{}_{1}=(ID_{A},ID_{B},TS_{A1},M_{OrdA1})$$

(29) $$h_{A}{}_{1}=H(M_{A}{}_{1})$$

Then, User A calculates the parameters of ECDSA, and uses the “Sign” of Algorithm 2 to generate the signature:

(30) $$(r_{A}{}_{1},s_{A}{}_{1})=Sign\left(k_{3},h_{A}{}_{1},d_{A}{}_{1}\right)$$

Afterward, User A uploads the order to the blockchain; in the meantime, it uses the public key $Puk_{B}$ of User B to encrypt a message $M_{A}{}_{1}$:

(31) $$Upload(M_{OrdA1},ID_{Order},h_{A}{}_{1},r_{A}{}_{1},s_{A}{}_{1})$$

(32) $$C_{A1}=E_{Puk_{{}_{B}}}(M_{A}{}_{1})$$

Finally, User A delivers $C_{A}{}_{1},\left(r_{A}{}_{1},s_{A}{}_{1}\right)$, which is A generated to User B.

Step 2. User B receives the message from User A and using its private key $Prk_{B}$ to decrypt $C_{A1}$ to acquire data $\left(ID_{A},ID_{B},TS_{A1},M_{OrdA1}\right)$ of $M_{A1}$, and verifies that the timestamp holds:

(33) $$\left(ID_{A},ID_{B},TS_{A1},M_{OrdA1})=D_{Prk_{B}}(C_{A1}\right)$$

(34) $$TS_{NOW}-TS_{BR1}\overset{?}{\leq }\Delta T$$

If Equation (34) is established, the smart contract “*Verify*” of Algorithm 2 is triggered to verify that the ECDSA signature is correct:

(35) $$h_{A1}{}^{\prime }=H(M_{A1})$$

(36) $$Verify\;\left(h_{A1}{}^{\prime },\;r_{A}{}_{1},s_{A}{}_{1}\right)$$

If it is correct, we can testify the message is from User A, and then User B generates a random number $k_{4}$ and uses order request information $M_{conf}$ and order information $M_{OrdA1}$ to generate a message $M_{B1}$. The message is sent to A and User B calculates $h_{B1}$:

(37) $$M_{B1}=(ID_{B},ID_{B},TS_{B1},M_{OrdA1},M_{conf})$$

(38) $$h_{B1}=H(M_{B1})$$

Then, User B calculates the parameters of the ECDSA and generates a signature by “Sign” of Algorithm 2:

(39) $$(r_{B}{}_{1},s_{B}{}_{1})=Sign\left(k_{4},h_{B}{}_{1},d_{B}{}_{1}\right)$$

Afterward, User A encrypts a message $M_{B}{}_{1}$ by the public key $Puk_{A}$ of User B:

(40) $$C_{B1}=E_{Puk_{{}_{A}}}(M_{B}{}_{1})$$

Finally, B sends $C_{B1},(r_{B}{}_{1},s_{B}{}_{1})$ to User A.

Step 3. User A receives the message from User B and uses his private key $Prk_{A}$ to decrypt $C_{B1}$ to acquire data $\left(ID_{B},ID_{B},TS_{B1},M_{OrdA1},M_{conf}\right)$ within the message $M_{B1}$, and verifies that the timestamp holds:
  (41) $$M_{B1}=(ID_{B},ID_{B},TS_{B1},M_{OrdA1},M_{conf})$$
  (42) $$TS_{NOW}-TS_{PR1}\overset{?}{\leq }\Delta T$$

If Equation (42) is established, the smart contract “*Verify*” of Algorithm 2 is triggered to verify the signature of ECDSA that is correct:

(43) $$h_{B1}{}^{\prime }=H(M_{B1})$$

(44) $$Verify\;\left(h_{B1}{}^{\prime },\;r_{B}{}_{1},s_{B}{}_{1}\right)$$

If it is correct, the message is proved to have been sent by User B. Otherwise, the order is voided. At this point, the order is confirmed.

**Figure 7:**
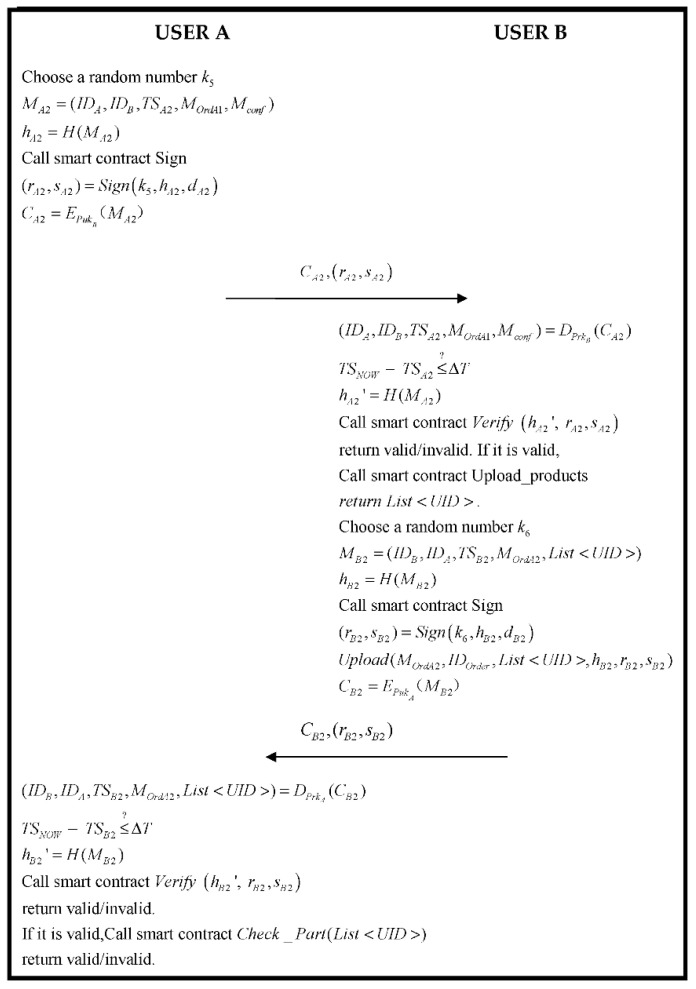
The flowchart of the transaction phase.

After the order phase mentioned above, both parties to the transaction have completed the task of placing and finalizing the order. In this phase, User B uploads the key information of the generated product to the blockchain. User A receives the product and information from User B and decrypts and verifies the correctness of the information. If it is accurate, the transaction is completed. The specific flowchart is as follows in Figure 7.

Step 1. User A generates a random number $k_{5}$, receives the product confirmation $M_{conf}$, and creates a message $M_{A}{}_{2}$. Calculating $h_{A}{}_{2}$:
  (45) $$M_{A}{}_{2}=(ID_{A},ID_{B},TS_{A2},M_{OrdA1},M_{conf})$$
  (46) $$h_{A}{}_{2}=H(M_{A}{}_{2})$$

Then, User A calculates the parameter of ECDSA and generates the signature by “Sign” of Algorithm 2:

(47) $$(r_{A}{}_{2},s_{A}{}_{2})=Sign\left(k_{5},h_{A}{}_{2},d_{A}{}_{2}\right)$$

After User A uses the public key $Puk_{B}$ of User B to encrypt $M_{A}{}_{1}$:

(48) $$C_{A2}=E_{Puk_{B}}(M_{A}{}_{2})$$

At last, User A sends $C_{A2},\left(r_{A}{}_{2},s_{A2}\right)$ to User B.

Step 2. User B receives the message from User A and using his private key $Prk_{B}$ decrypts $C_{A1}$ to acquire the data $\left(ID_{A},ID_{B},TS_{A2},M_{OrdA1},M_{conf}\right)$ within $M_{A2}$, in the meantime verifying if the timestamp is legal:

(49) $$\left(ID_{A},ID_{B},TS_{A2},M_{OrdA1},M_{conf})=D_{Prk_{B}}(C_{A2}\right)$$

(50) $$TS_{NOW}-TS_{A2}\overset{?}{\leq }\Delta T$$

If (50) is established, the smart contract “*Verify*” of Algorithm 2 is triggered to verify that the signature of ECDSA is correct:

(51) $$h_{A2}{}^{\prime }=H(M_{A2})$$

(52) $$Verify\;\left(h_{A2}{}^{\prime },\;r_{A2},s_{A2}\right)$$

If Equation (52) is correct, it proves that the order information is sent by User A, triggering smart contacts *UploadParts* or *UploadVehicles* within Algorithm 3 or Algorithm 4 to upload the information of products. If it is a transaction among AM, RS, and PM, UploadParts is triggered, and if it is a transaction between CD and AM, UploadVehicles is triggered. In the meantime, the functions List<UID> (UID symbol $ID_{Car}$ or $ID_{Part}$). Then, User B generates a random number $k_{6}$ and uses List<UID>, and $Order_{A1}$ generates $M_{B1}$, which is returned with information of the order. Calculating $h_{B1}$:

(53) $$M_{B2}=(ID_{B},ID_{A},TS_{B2},M_{OrdA2},List<UID>)$$

(54) $$h_{B2}=H(M_{B2})$$

Then, User B calculates the parameter of ECDSA and generates a signature by “Sign” of Algorithm 2.

**Algorithm 3:** Chaincode UploadParts of the proposed schemevar PI []PartInfofunc UploadParts(var pnum int, var PUID string, var PName string, var PParameter string, var PAgingStandard string, var PManuName string, var PProductionDate string, var PExfactoryDate string, var PAging bool){        for (*i = 0; i < pnum; i++*){                PI = append(PI, new PartInfo{                PUID: PUID[*i*]                PName: PName                PParameter: PParameter                PAgingStandard: PAgingStandard                PManuName: PManuName                PProductionDate: PProductionDate[*i*]                PExfactoryDate: time.Now                PAging: false})                ListPUIDs = append(ListPUIDs, PI[*i*].ListPUIDs)                return ListPUIDs        } }

Step 3. User A acquires the message of User B, uses his private key $Prk_{A}$ decrypting $C_{B2}$ to obtain data $\left(ID_{B},ID_{A},TS_{B2},M_{OrdA2},List<UID>\right)$ within $M_{B2}$, and verifies if the timestamp is correct:

(55) $$\left(ID_{B},ID_{A},TS_{B2},M_{OrdA2},List<UID>)=D_{Prk_{A}}(C_{B2}\right)$$

(56) $$TS_{NOW}-TS_{B2}\overset{?}{\leq }\Delta T$$

If the verification passes the above, if the above verification holds, “*Verify*” of Algorithm 2 is triggered and checking if the signature of ECDSA is correct:

(57) $$h_{B2}{}^{\prime }=H(M_{B2})$$

(58) $$Verify\;\left(h_{B2}{}^{\prime },\;r_{B}{}_{2},s_{B}{}_{2}\right)$$

If it is true, the system triggers the smart contract Algorithm 5 and proves the information of the product. If it is successful, the transaction finishes.

**Algorithm 4:** Chaincode UploadVehicles of the proposed schemevar VI []VehicleInfo func UploadVehicles(var num int, var VUID string, var VName string, var VParameter string, var VAgingStandard string, var VManuName string, var VProductionDate string, var VExfactoryDate string, var VAging bool, var VPUIDs []string){              for *(i = 0; i < vnum; i++*){              VI = append(VI, new VehicleInfo{              VUID: VUID[*i*]              VName: VName              VParameter: VParameter              VAgingStandard: VAgingStandard              VManuName: VManuName              VProductionDate: VProductionDate[i]              VExfactoryDate: time.Now              VAging: false              for(*j = 0; i < pnum;j++*){                      VPUIDs[*j*]: VPUIDs[*j*]   }              })              ListVUIDs = append(ListVUIDs, VI[i].ListVUIDs)              return ListVUIDs        } } 

**Algorithm 5:** Chaincode Check_products of the proposed schemefunc CheckParts(var pnum int. ListPUIDs []string){ for(*i = 0; i < pnum; i++*){        if(PI[*i*].PAging == True)                return “invalid”    }        return “valid” } func CheckVehicles(var vnum int. ListVUIDs []string){        index = searchCar(VI, VUID)        if(index ! = null)                return “invalid”        for(*i = 0; i < vnum; i++*){                index2 = searchPID()                if(PI[*i*].PAging == True)        return “invalid”    }                return “valid” } 

### 3.6. Sale Phase

**Figure 8:**
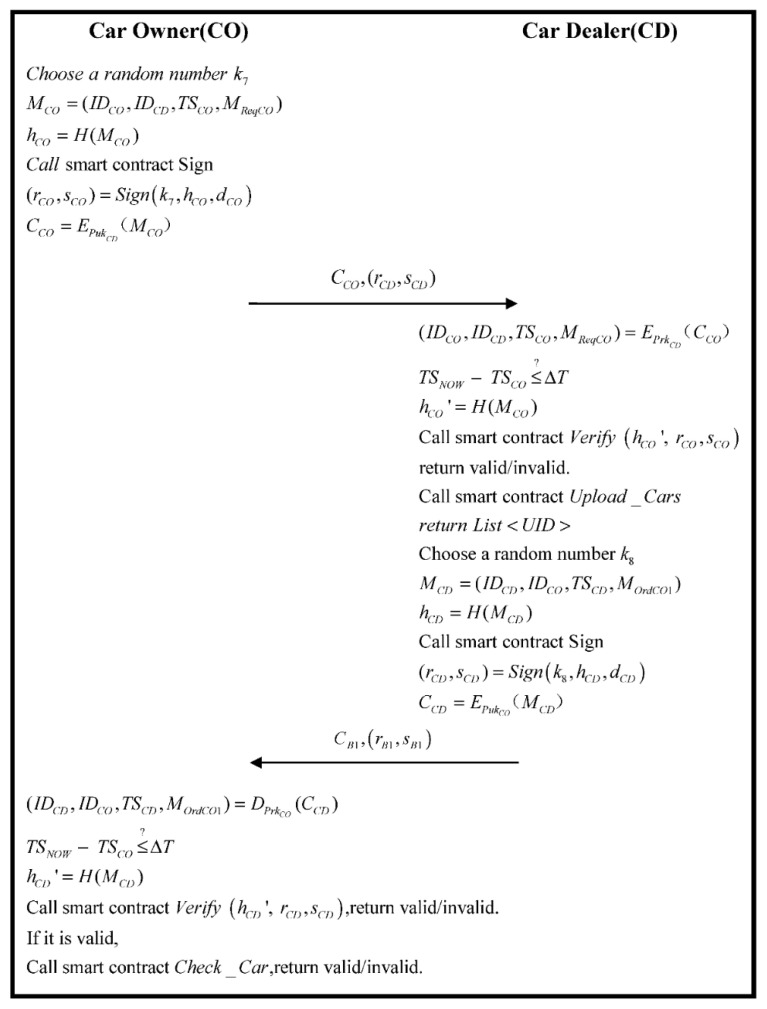
The flowchart of the sale phase.

In the phase, CO purchases vehicle in the CD. The specific process is as following Figure 8.

Step 1. CO choices a product and sends $M_{ReqCO}$ to a CD. First, CO generates a random number $k_{7}$ and generates $M_{CO}$. Calculating $h_{CO}$:

(59) $$M_{CO}=(ID_{CO},ID_{CD},TS_{CO},M_{ReqCO})$$

(60) $$h_{CO}=H(M_{CO})$$

Then, CO calculates the parameter of ECDSA and generates a signature by “Sign” of Algorithm 2, and uses the public key of CD to encrypt:

(61) $$(r_{CO},s_{CO})=Sign\left(k_{7},h_{CO},d_{CO}\right)$$

(62) $$C_{CO}=E_{Puk_{{}_{CD}}}(M_{CO})$$

At last, CO sends $C_{CO},\left(r_{CO},s_{CO}\right)$ to CD.

Step 2. CD receives data $\left(ID_{CO},ID_{CO},TS_{CO},M_{ReqCO}\right)$ from $C_{CO}$, and verifies if the timestamp is correct:

(63) $$\left(ID_{CO},ID_{CD},TS_{CO},M_{ReqCO})=E_{Prk_{{}_{CD}}}(C_{CO}\right)$$

(64) $$TS_{NOW}-TS_{CO}\overset{?}{\leq }\Delta T$$

If (74) is correct, the smart contract “*Verify*” of Algorithm 2 is triggered to verify if the signature of ECDSA is legal or not:

(65) $$h_{CO}{}^{\prime }=H(M_{CO})$$

(66) $$Verify\;\left(h_{CO}{}^{\prime },\;r_{CO},s_{CO}\right)$$

If it is true, it proves the information of the order that sends from CO. Additionally, the system finds the vehicle of the request of the order. CD sends $ID_{Car}$ to CO and a random number $k_{8}$ is generated by CD. In the meantime, according to $UID_{part}$ and $M_{OrdCO1}$, which are created by CO, message $M_{CD}$ is generated. Returning the information of the order to CO calculate $h_{CD}$:

(67) $$M_{CD}=(ID_{CD},ID_{CO},TS_{CD},M_{OrdCO1})$$

(68) $$h_{CD}=H(M_{CD})$$

Additionally, then CD calculates the parameter of ECDSA and generates a signature by “Sign” of Algorithm 2:

(69) $$(r_{CD},s_{CD})=Sign\left(k_{8},h_{CD},d_{CD}\right)$$

Afterward, the CD using the public key $Puk_{CO}$ of CO encrypts $M_{CD}$:

(70) $$C_{CD}=E_{Puk_{CO}}(M_{CD})$$

At last, CD sends $C_{CD},(r_{CD},s_{CD})$ to CO.

Step 3. CO receiving the message from CD, using its private key $Prk_{CO}$, decrypts $C_{CD}$ to acquire data $\left(ID_{CD},ID_{CO},TS_{CD},M_{OrdCO1}\right)$ within $M_{CD}$, and it verifies if the timestamp is correct:

(71) $$\left(ID_{CD},ID_{CO},TS_{CD},M_{OrdCO1})=D_{Prk_{CO}}(C_{CD}\right)$$

(72) $$TS_{NOW}-TS_{CO}\overset{?}{\leq }\Delta T_{\left(2\right)}$$

If (72) is established, the smart contract “*Verify*” of Algorithm 2 is triggered, in the meantime verifying if the signature of ECDSA is correct or not:

(73) $$h_{CD}{}^{\prime }=H(M_{CD})$$

(74) $$Verify\;\left(h_{CD}{}^{\prime },\;r_{CD},s_{CD}\right)$$

If it is correct, Algorithm 6 is triggered, and the transaction is finished.

### 3.7. Repair Phase

**Figure 9:**
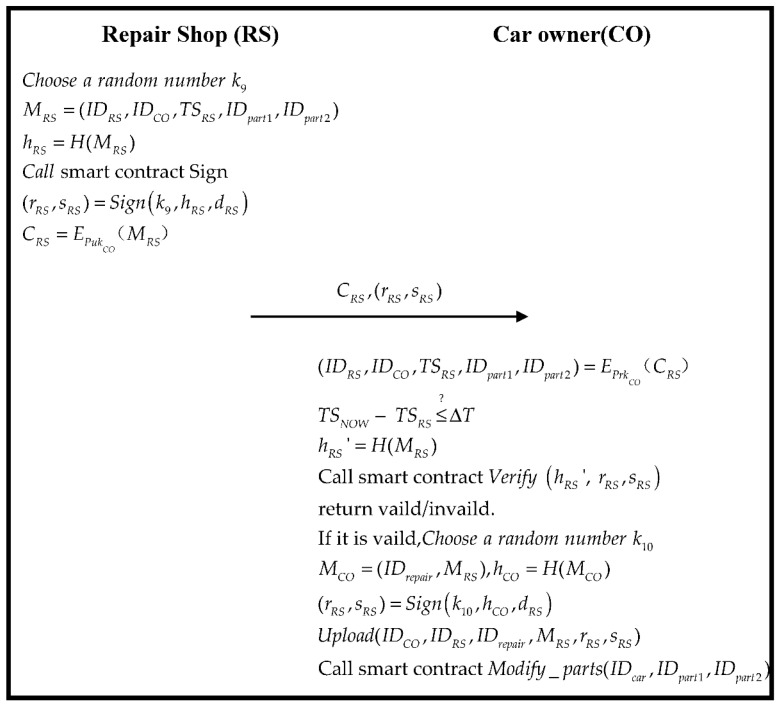
The flowchart of the repair phase.

At this stage, CO goes to RS for vehicle maintenance. The specific process is shown in Figure 9.

Step 1. RS sends $ID_{part1}$ of the old parts and $ID_{part2}$ of new parts that need to be replaced to the CO, and generates random numbers $k_{9}$:

(75) $$M_{RS}=(ID_{RS},ID_{CO},TS_{RS},ID_{part1},ID_{part2})$$

(76) $$h_{RS}=H(M_{RS})$$

Then, the user CO calculates the parameters of ECDSA, generates a signature through “Sign” of Algorithm 2, and then encrypts it with the CO’s public key:

(77) $$(r_{RS},s_{RS})=Sign\left(k_{9},h_{RS},d_{RS}\right)$$

(78) $$C_{RS}=E_{Puk_{{}_{CO}}}(M_{RS})$$

Finally, RS sends $C_{RS},(r_{RS},s_{RS})$, which is generated and sent to CO.

Step 2. CO receives the data $\left(ID_{RS},ID_{CO},TS_{RS},ID_{part1},ID_{part2}\right)$ of the message $M_{RS}$ from RS and verifies whether the timestamp holds:

(79) $$\left(ID_{RS},ID_{CO},TS_{RS},ID_{part1},ID_{part2})=E_{Prk_{{}_{CO}}}(C_{RS}\right)$$

(80) $$TS_{NOW}-TS_{RS}\overset{?}{\leq }\Delta T$$

If established, it triggers the smart contract “*Verify*” of Algorithm 2 to verify that the ECDSA signature is correct:

(81) $$h_{RS}{}^{\prime }=H(M_{RS})$$

(82) $$Verify\;\left(h_{RS}{}^{\prime },\;r_{RS},s_{RS}\right)$$

If the verification is passed, a random number $k_{10}$ is generated after confirming the information $M_{CO}$, a message is generated, and then the maintenance message is signed and uploaded.

(83) $$M_{CO}=(ID_{repair},M_{RS})$$

(84) $$h_{CO}=H(M_{CO})$$

(85) $$(r_{RS},s_{RS})=Sign\left(k_{10},h_{CO},d_{RS}\right)$$

(86) $$Upload(ID_{CO},ID_{RS},ID_{repair},M_{RS},r_{RS},s_{RS})$$

Trigger the smart contract after uploading Algorithm 6.

**Algorithm 6:** Chaincode Modify_parts of the proposed schemefunc ModifyPart(var VUID string, var newPUID string, var oldPUID string){        index = searchCar(VI, VUID)        if(index! = null)                index2 = searchVheiclePUIDs(VI[index].VehiclePUIDs,oldPUID)                 if(index2! = null)                        replace(VI[index].VehiclePUID[index2],newPUID)                        index3 = searchPUID(PI,oldPUID)                        PI[index]. Paging = True                        return “valid”                else                        return ”invalid”        else                return ”invalid” } 

### 3.8. Arbitration Phase

**Figure 10:**
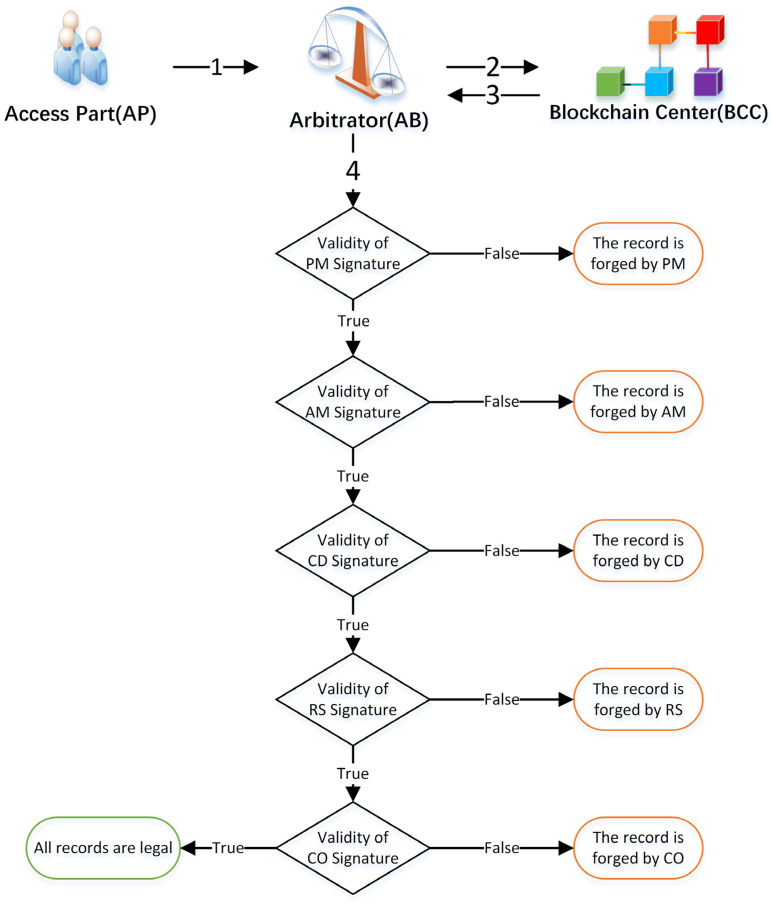
The validation flow in the arbitration phase.

When either party doubts the validity of a part, they can arbitrate its legitimacy through Arbitration. The process of arbitration is shown in Figure 10, and the numbers 1–4 correspond to step 1–4. The precise details of this process are as follows:

Step 1. AP provides the UID of a specific part to AB.
Step 2. AB sends a TID request message with its signature to BCC.
Step 3. BCC checks the signature of AB, and if the signature is valid, BBC delivers the signature list to AB.
Step 4. AB checks the validity of each signature in the signature list. The order of the checks is as follows.
  (a) Verify the signature of PM, if it is not legal, the record is proved to be forged by PM.
  (b) Otherwise Verify the signature of AM, if it is not legitimate, the record is forged by AM.
  (c) Verify the signature of the CD, if it is not legal, the record is proved to be forged by the CD.
  (d) Verify the signature of RS, if it is not legal, the record is proved to be forged by RS.
  (e) Verify the signature of CO, if it is not legal, the record is proved to be forged by CO.
  (f) If all the above signature is valid, then the process of circulation of the part is proven and verified by AU.

## 4. Analysis

### 4.1. Data Integrity

We use ECDSA and hash functions to ensure data integrity. In a blockchain, each participant has a pair of public and private keys. The sender must compute a hash and generate a set of signatures using the receiver’s public key before sending the message, and the receiver needs to verify the message and the signatures using his private key to ensure the validity of the message. If the attacker tampers with the data to send to the receiver, then the receiver will verify if the hash value and signature are not passed. All phases’ detailed information is listed in Table 2.

### 4.2. Non-Repudiation

In this paper, we use Verify of ECDSA to resolve the repudiation issue. In the blockchain mechanism, all messages transmitted by the sender must sign with their private key, and the receiver using the sender’s public key verifies the messages. That ensures messages cannot be denied. Table 3 is the non-repudiation verification of the proposed scheme.

### 4.3. Traceability and Unforgeability

Based on blockchain characteristics, we learn that all transaction records are stored and chained to the ledger of every peer, and the records are traceable and unforgeable. In the meantime, data can be verified and transparent. For example, AB can trace records to verify whether blockchain data are legal or not. In Figure 10, if the signature cannot pass the verification, the signatures are forged.

### 4.4. Man-in-the-Middle Attack

Man-in-the-middle attack (MIMT) generally refers to the attacker intercepting the normal network communication data between the client and the server [33]. In the communication protocol, each communication message on the blockchain uses asymmetric encryption for defense against MIMT, i.e., the receiver’s public key encrypts the message when it is sent, and the receiver decrypts the message with his or her private key to ensure that the source of the message is correct.

Scenario: An attacker tampers with the communication messages or eavesdrops between the communicating parties.

Analysis: In the blockchain, the sender uses the public key of the receiver to encrypt messages. Additionally, if the attacker did not use a match private key to decrypt, it did not learn the content of the message. The private key only is known to the receiver.

For example, in the authentication phase, User A encrypts the message $M_{A}{}_{1}$ with User B’s public key $Puk_{B}$, then generates a ciphertext $C_{A1}$ and sends it to User B. B then uses his private key $Prk_{B}$ to decrypt the ciphertext to obtain the original message $M_{A}{}_{1}$. The related details are shown as follows:

(87) $$C_{A1}=E_{Puk_{B}}(M_{A}{}_{1})$$

(88) $$M_{A1}=D_{Prk_{B}}(C_{A1})$$

Therefore, it is guaranteed that the attacker cannot decrypt the message without the receiver’s private key. Each stage of asymmetric encryption and decryption is shown in Table 4.

### 4.5. Replay Attack

A replay attack is a type of network attack that uses malicious or fraudulent ways to repeat or delay valid data and the attacker intercepts the message of the communication and retransmits the data to the receiver [34]. In this study, to prevent the replay attack, we add a timestamp to each message, and the receiver needs to calculate the difference of the timestamp when receiving the corresponding message and compare it with the set threshold value, and if the time difference exceeds the threshold value it identifies that the message is being replayed.

Scenario: An attracter listens to messages between sender and receiver and, after that, it re-sends the same message to the receiver.

Analysis: If the receiver receives the ciphertext and decrypts it to acquire the timestamp $TS_{X}$ of the sender, the receiver verifies that the difference between the current timestamp $TS_{NOW}$ and the timestamp in the message is less than a threshold ΔT. When this does not hold, the communication that suffered a replay attack is confirmed.

For example, in the verification phase, the timestamp $TS_{A1}$ when User A sends the data will be detected $TS_{NOW}-TS_{A1}\overset{?}{\leq }\Delta T$ when User B receives the data, and if it passes, it proves that the data are not under replay attack. Table 5 is the timestamp verification for each stage, where the timestamp after the receiver receives the data is collectively called.

### 4.6. Counterfeiting Attack

In this paper, the counterfeiting attack is the behavior of an attacker using falsified and uploaded fake parts’ information or disguising as a parts owner to trade on the system. We verify the legitimacy of the data during the transaction process to prevent this attack.

Scenario 1: The attacker fakes and uploads fake parts’ information, and uses these parts to trade.

Analysis 1: Uploading parts’ information is a unique function of PM. Other users cannot sign and upload parts without a PM private key. Additionally, because the alliance chain is used, each role needs to be authenticated, and the chances of an attacker disguising PM successfully are not possible. At the same time, based on the characteristics of the blockchain, the source of the parts can be traced. Therefore, when that counterfeit part appears on the blockchain, we can quickly locate the attacker.

Scenario 2: Malicious RS or rental car users replace expensive parts with low-cost fake parts.

**Figure 11:**
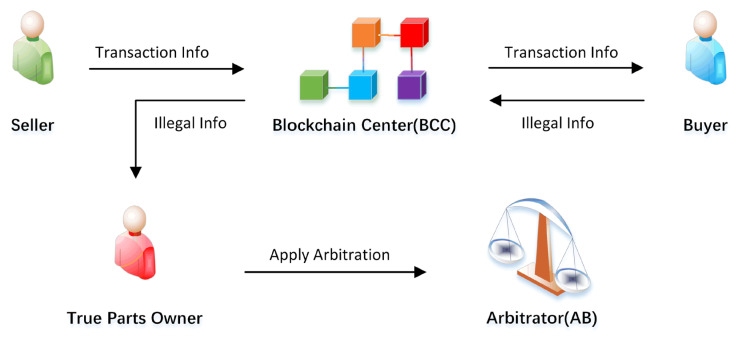
Trading illegal parts handling process.

Analysis 2: In our proposal, the part and the vehicle to which it belongs are bound together and belong to the same owner. As shown in Figure 11, when malicious RS replaced expensive parts reappear on the supply chain and conduct transactions, the buyer of the part will check again whether the source of the part is legitimate. If not, the system will notify the original owner of the part, who can quickly apply for arbitration with an arbitration institution.

## 5. Discussion

### 5.1. Communication Cost

In this section, we calculate the different communication costs for different network rates as shown in Table 6. Firstly, we assume that the length of the ECDSA key and signature is 160 bits, the length of asymmetrically encrypted data is 1024 bits, and other information (ID, timestamp, etc.) is 80 bits. The total size is 160 bits × 2 + 80 bits × 2 + 1024 bits × 2 = 2588 bits. It takes 0.431 ms in 3G (6 Mpbs), communication environment, 0.026 ms in 4G (100 Mpbs) communication environment, and 0.129 us in 5G (20 Gpbs) communication environment [35].

### 5.2. Computation Cost

Table 7 shows the computational cost analysis of the roles in each phase. Taking the authentication phase as an example, in this phase both User A and User B need to perform the signature operation, verification operation, encryption, and decryption operation, comparison operation once each, and hash operation twice.

### 5.3. Function Comparison

Table 8 shows the comparison with the previous researchers. In this paper, we proposed a blockchain-based automotive and parts supply chain service framework, related algorithm, and communication protocol and analyzed related cost and security.

## 6. Conclusions

The quality of vehicles and parts is closely related to traffic safety. To solve safety hazards caused by flaws in vehicles and parts and information asymmetry between providers and consumers, we proposed an automotive supply chain framework that is based on blockchain and smart contracts, in the meantime also designing communication flows and algorithms in the blockchain. In our analysis and discussion, this study-proposed system has excellent performance and security.

In this blockchain system, all access parties must register with BC to require a pair of public-private keys and a unique ID; in the meantime, both communicating parties should authenticate each other’s identities before communicating. In addition, during communication, each role signs and encrypts the information to be sent and uploads it to the chain, and decrypts and verifies the validity of the received message. Furthermore, when a dispute arises with a participant in the system, the participant can apply for arbitration by AB. Additionally, then AB, using the participant, provides a message to acquire blockchain information, confirming the legality.

By the proposed method and framework, we accomplish the features as follows:

(1) Proposed a completely auto supply framework based-blockchain.
(2) Using asymmetrical encryption/decryption to ensure data integrity.
(3) Design some algorithms for simple quality identification of cars and parts.
(4) Analyzing costs of computation and communication.
(5) Parties can verify the legality of an asset by an arbitrator.
(6) Simulate defense against known attacks.

## Figures and Tables

**Table 2:** Verification of the data integrity of the proposed scheme.

Phase	Party		Message	Hash Value	Verification
	Sender	Receiver			
Authentication	USER A	USER B	$M_{A}{}_{1}=(ID_{A},ID_{B},TS_{A1},Info_{A})$	$h_{A1}=H(M_{A}{}_{1})$	$Verify(h_{A1},r_{A1},s_{A1})$
	USER B	USER A	$M_{B}{}_{1}=(ID_{B},ID_{A},TS_{B1},Info_{B})$	$h_{B1}=H(M_{B1})$	$Verify(h_{B1},r_{B1},s_{B1})$
Order and Transaction phase	USER A	USER B	$M_{A}{}_{1}=(ID_{A},ID_{B},TS_{A1},Order_{A1})$	$h_{A}{}_{1}=H(M_{A}{}_{1})$	$Verify\left(h_{A1}{}^{\prime },r_{A}{}_{1},s_{A}{}_{1}\right)$
	USER B	USER A	$M_{B1}=(ID_{B},ID_{B},TS_{B1},M_{Ord}{}_{A1},M_{conf})$	$h_{B1}=H(M_{B1})$	$Verify\;\left(h_{B1}{}^{\prime },\;r_{B}{}_{1},s_{B}{}_{1}\right)$
	USER A	USER B	$M_{A}{}_{2}=(ID_{A},ID_{B},TS_{A2},Order_{A1},Info_{Confirm})$	$h_{A}{}_{2}=H(M_{A}{}_{2})$	$Verify\;\left(h_{A2}{}^{\prime },\;r_{A2},s_{A2}\right)$
	USER B	USER A	$M_{B2}=(ID_{B},ID_{A},TS_{B2},Order_{A2},List<UID>)$	$h_{B2}=H(M_{B2})$	$Verify\left(h_{B2}{}^{\prime },r_{B}{}_{2},s_{B}{}_{2}\right)$
Sale phase	Car Owner (CO)	Car Dealer (CO)	$M_{CO}=(ID_{CO},ID_{CD},TS_{CO},Request)$	$h_{CO}=H(M_{CO})$	$Verify\left(h_{CO}{}^{\prime },r_{CO},s_{CO}\right)$
	Car Dealer (CD)	Car Owner (CD)	$M_{CD}=(ID_{CD},ID_{CO},TS_{CD},Order_{CO})$	$h_{CD}=H(M_{CD})$	$Verify\;\left(h_{CD}{}^{\prime },\;r_{CD},s_{CD}\right)$
Repair phase	Repair Shop	Car Owner (CO)	$M_{RS}=(ID_{RS},ID_{CO},TS_{RS},ID_{part_{old}},ID_{part_{new}})$	$h_{RS}=H(M_{RS})$	$Verify\;\left(h_{RS}{}^{\prime },\;r_{RS},s_{RS}\right)$

**Table 3:** Non-repudiation verification of the proposed scheme.

Phase	Party		Message	Signature	Verification
	Sender	Receiver			
Authentication	USER A	USER B	$M_{A}{}_{1}=(ID_{A},ID_{B},TS_{A1},Info_{A})$	$Sign(k_{1},h_{A1},d_{A})$	$Verify(h_{A1},r_{A1},s_{A1})$
	USER B	USER A	$M_{B}{}_{1}=(ID_{B},ID_{A},TS_{B1},Info_{B})$	$Sign(k_{2},h_{B1},d_{B})$	$Verify(h_{B1},r_{B1},s_{B1})$
Order and Transaction phase	USER A	USER B	$M_{A}{}_{1}=(ID_{A},ID_{B},TS_{A1},Order_{A1})$	$Sign\left(k_{3},h_{A}{}_{1},d_{A}{}_{1}\right)$	$Verify\left(h_{A1}{}^{\prime },r_{A}{}_{1},s_{A}{}_{1}\right)$
	USER B	USER A	$M_{B1}=(ID_{B},ID_{B},TS_{B1},Order_{A1},Info_{Confirm})$	$Sign\left(k_{4},h_{B}{}_{1},d_{B}{}_{1}\right)$	$Verify\;\left(h_{B1}{}^{\prime },\;r_{B}{}_{1},s_{B}{}_{1}\right)$
	USER A	USER B	$M_{A}{}_{2}=(ID_{A},ID_{B},TS_{A2},Order_{A1},Info_{Confirm})$	$Sign\left(k_{5},h_{A}{}_{2},d_{A}{}_{2}\right)$	$Verify\;\left(h_{A2}{}^{\prime },\;r_{A2},s_{A2}\right)$
	USER B	USER A	$M_{B2}=(ID_{B},ID_{A},TS_{B2},Order_{A2},List<UID>)$	$Sign\left(k_{6},h_{B2},d_{B}{}_{2}\right)$	$Verify\left(h_{B2}{}^{\prime },r_{B}{}_{2},s_{B}{}_{2}\right)$
Sale phase	Car Owner (CO)	Car Dealer (CO)	$M_{CO}=(ID_{CO},ID_{CD},TS_{CO},Request)$	$Sign\left(k_{7},h_{CO},d_{CO}\right)$	$Verify\left(h_{CO}{}^{\prime },r_{CO},s_{CO}\right)$
	Car Dealer (CD)	Car Owner (CD)	$M_{CD}=(ID_{CD},ID_{CO},TS_{CD},Order_{CO})$	$Sign\left(k_{8},h_{CD},d_{CD}\right)$	$Verify\;\left(h_{CD}{}^{\prime },\;r_{CD},s_{CD}\right)$
Repair phase	Repair Shop	Car Owner (CO)	$M_{RS}=(ID_{RS},ID_{CO},TS_{RS},ID_{part_{old}},ID_{part_{new}})$	$Sign\left(k_{9},h_{RS},d_{RS}\right)$	$\;Verify\;\left(h_{RS}{}^{\prime },\;r_{RS},s_{RS}\right)$

**Table 4:** Encryption and decryption to prevent a man-in-the-middle attack.

Phase	Party		Encryption	Decryption
	Sender	Receiver		
Authentication	USER A	USER B	$C_{A1}=E_{Puk_{B}}(M_{A}{}_{1})$	$M_{A1}=D_{Prk_{B}}(C_{A1})$
	USER B	USER A	$C_{B}{}_{1}=E_{Puk_{A}}(M_{B}{}_{1})$	$M_{B1}=D_{Prk_{A}}(C_{B1})$
Order	USER A	USER B	$C_{A}{}_{1}=E_{Puk_{{}_{B}}}(M_{A}{}_{1})$	$M_{A}{}_{1}=D_{Prk_{B}}(C_{A1})$
	USER B	USER A	$C_{B}{}_{1}=E_{Puk_{{}_{A}}}(M_{B}{}_{1})$	$M_{B}{}_{1}=D_{Prk_{A}}(C_{B1})$
Transaction	USER A	USER B	$C_{A}{}_{2}=E_{Puk_{{}_{B}}}(M_{A}{}_{2})$	$M_{A}{}_{2}=D_{Prk_{B}}(C_{A2})$
	USER B	USER A	$C_{B}{}_{2}=E_{Puk_{{}_{A}}}(M_{B}{}_{2})$	$M_{B}{}_{2}=D_{Prk_{A}}(C_{B2})$
Sale	Car Owner (CO)	Car Dealer (CO)	$C_{CO}=E_{Puk_{{}_{CD}}}(M_{CO})$	$M_{CO}=D_{Prk_{{}_{CD}}}(C_{CO})$
	Car Dealer (CD)	Car Owner (CD)	$C_{CD}=E_{Puk_{CO}}(M_{CD})$	$M_{CD}=D_{Prk_{CO}}(C_{CD})$
Repair	Repair Shop	Car Owner (CO)	$C_{RS}=E_{Puk_{{}_{CO}}}(M_{RS})$	$M_{RS}=D_{Prk_{{}_{CO}}}(C_{RS})$

**Table 5:** Timestamp validation to prevent replay attack.

Phase	Party		Send Time	Validation
	Sender	Receiver		
Authentication	USER A	USER B	$TS_{A1}$	$TS_{NOW}-TS_{A1}\overset{?}{\leq }\Delta T$
	USER B	USER A	$TS_{B1}$	$TS_{NOW}-TS_{B1}\overset{?}{\leq }\Delta T$
Order and Transaction	USER A	USER B	$TS_{A1}$	$TS_{NOW}-TS_{A1}\overset{?}{\leq }\Delta T$
	USER B	USER A	$TS_{B1}$	$TS_{NOW}-TS_{B1}\overset{?}{\leq }\Delta T$
	USER A	USER B	$TS_{A2}$	$TS_{NOW}-TS_{A2}\overset{?}{\leq }\Delta T$
	USER B	USER A	$TS_{B2}$	$TS_{NOW}-TS_{B2}\overset{?}{\leq }\Delta T$
Sale	Car Owner (CO)	Car Dealer (CO)	$TS_{CO}$	$TS_{NOW}-TS_{CO}\overset{?}{\leq }\Delta T$
	Car Dealer (CD)	Car Owner (CD)	$TS_{CD}$	$TS_{NOW}-TS_{CO}\overset{?}{\leq }\Delta T$
Repair	Repair Shop	Car Owner (CO)	$TS_{RS}$	$TS_{NOW}-TS_{RS}\overset{?}{\leq }\Delta T$

**Table 6:** Communication costs of the proposed scheme.

Phase	Message Length	3G (6 M bps)	4G (100 M bps)	5G (20 G bps)
Authentication	2588 bits	0.431 ms	0.026 ms	0.129 us
Order	2588 bits	0.431 ms	0.026 ms	0.129 us
Transaction	2588 bits	0.431 ms	0.026 ms	0.129 us
Sale	2588 bits	0.431 ms	0.026 ms	0.129 us
Repair	1294 bits	0.216 ms	0.013 ms	0.065 us

**Table 7:** Computation costs of the proposed scheme.

Phase	Access Part 1	Access Part 2
Authentication	User A	User B
	$T_{sig}+T_{ver}+2T_{hash}+T_{cmp}+2T_{E/D}$	$T_{sig}+T_{ver}+2T_{hash}+T_{cmp}+2T_{E/D}$
Order	User A	User B
	$T_{sig}+T_{ver}+T_{upload}+T_{cmp}+2T_{E/D}+2T_{hash}$	$T_{sig}+T_{ver}+2T_{hash}+T_{cmp}+2T_{E/D}$
Transaction	User A	User B
	$T_{sig}+T_{ver}+2T_{hash}+T_{cmp}+2T_{E/D}+T_{chd}$	$T_{sig}+T_{ver}+2T_{hash}+T_{cmp}+2T_{E/D}+T_{upload}$
Sale	Car Owner( CO)	Car Dealer (CO)
	$T_{sig}+T_{ver}+2T_{hash}+T_{cmp}+2T_{E/D}+T_{chd}$	$T_{sig}+T_{ver}+2T_{hash}+T_{cmp}+2T_{E/D}$
Repair	Repair Shop (RS)	Car Owner (CO)
	$T_{sig}+T_{hash}+T_{E/D}$	$T_{sig}+T_{hash}+T_{E/D}$

Note: $T_{sig}$: Signature operation; $T_{ver}$: Verify operation; $T_{E/D}$: Encryption/Decryption operation; $T_{hash}$: Hash function operation; $T_{cmp}$: Comparison operation; $T_{chd}$: Check data function; $T_{upload}$: Upload data operation.

**Table 8:** Comparison with surveyed related works.

Authors	Year	Objectives	1	2	3	4	5
Chen et al. [16]	2015	A theoretical framework for combining blockchain and supply chain	N	Y	N	Y	N
Sharma et al. [17]	2018	A distributed framework model for the entire life cycle phases of the automotive industry blockchain-based	N	Y	Y	Y	Y
Kim et al. [18]	2019	A blockchain-based design for authentication of parts	N	Y	N	Y	N
Miehle et al. [21]	2019	A traceable parts supply chain application built on blockchain and smart contracts	N	Y	N	Y	N
Helo and Hao [22]	2019	A Blockchain-based logistics monitoring system prototype	N	Y	N	Y	Y
Yahiaoui et al. [23]	2020	Blockchain and smart contract-based supply chain model	N	Y	N	Y	N
Li and Ye [24]	2020	Combines blockchain and ASC for distributed storage of production and sales data	N	Y	N	Y	N
Wang et al. [25]	2020	Blockchain-based Product-Service System service framework for vehicle products	N	Y	N	Y	Y
Our method	2022	Blockchain-based ASC and service framework	Y	Y	Y	Y	Y

Notes: 1: Communication protocol, 2: Blockchain-based architecture, 3: Algorithm, 4: Complete architecture or framework, 5: Analysis, Y: Yes, N: No.

## Data Availability

The data used to support the findings of this study are available from the corresponding author upon request.

## Notations

q	A k bit prime number
$\begin{array}{c} GF\left(q\right) \end{array}$	Finite group of *q*
E	Elliptic Curves Defined on Finite Groups *q*
G	A generating points based on Elliptic Curve *E*
$k_{i}$	The *i*th random value on the elliptic curve
$d_{X}/Q_{X}$	The ECDSA’s private key/public key of the party *X*
$\left(x_{Ai},y_{Ai}\right)/\left(r_{Ai},s_{Ai}\right)$	The *i*th ECDSA/Elliptic curve signature value of User *A*
$TS_{Xi}/TS_{NOW}$	The *i*th timestamp of *X*/current timestamp
$M_{Xi}$	The *i*th message is generated by *X*
$M_{Info_{X}}/M_{BC_{X}}$	User Info of *X*/Blockchain Message for *X*
$M_{Conf}/M_{OrdXi}$	order Confirmation/The *i*th order information from *X*
$C_{Xi}$	The *i*th encrypted ciphertext is generated by *X*
$ID_{X}$	Unique ID of User *X*
$ID_{Car}/ID_{Part}/ID_{Order}/ID_{repair}$	Unique identification code of the vehicle/part/Order/Repair
H(M)	One-way hash function
$h_{Xi}$	The *i*th hash value of X
$Puk_{X}/Prk_{X}$	X own public/private key that issued by BCC
$E_{Puk_{X}}(M)/D_{Prk_{X}}(M)$	Encrypt/Decrypt message M using X public/private key
$A\overset{?}{=}B/A\overset{?}{\leq }B$	Verify that A is equal to B/Check if A is less than B
